# P-948. Performance Characteristics of MRSA Nares PCR in Pediatric Lower Respiratory Tract Infections: A Prospective Evaluation

**DOI:** 10.1093/ofid/ofaf695.1151

**Published:** 2026-01-11

**Authors:** Zac Aldewereld, Michael D Green, Hannah M Creager, Ruby Sangha, Evan E Facer

**Affiliations:** UPMC Children's Hospital of Pittsburgh, Pittsburgh, Pennsylvania; University of Pittsburgh School of Medicine, Pittsburgh, PA; University of Pittsburgh Medical Center, Pittsburgh, Pennsylvania; UPMC Childrens Hospital of Pittsburgh, Pittsburgh, PA; St. Louis Children's Hospital, St. Louis, Missouri

## Abstract

**Background:**

Inappropriate antibiotic use contributes to antimicrobial resistance and adverse drug events. Empiric anti-MRSA therapy is commonly used in pediatric lower respiratory tract infections (LRTIs), though often unnecessarily. While retrospective studies have shown MRSA nasal swabs have high negative predictive value (NPV), prospective pediatric data remain limited. This study evaluates the diagnostic performance of MRSA nares PCR in hospitalized children with suspected LRTIs.

Summary of Patient-Level Data and Test Characteristics for MRSA Nares PCR Evaluation

**Table 1.**
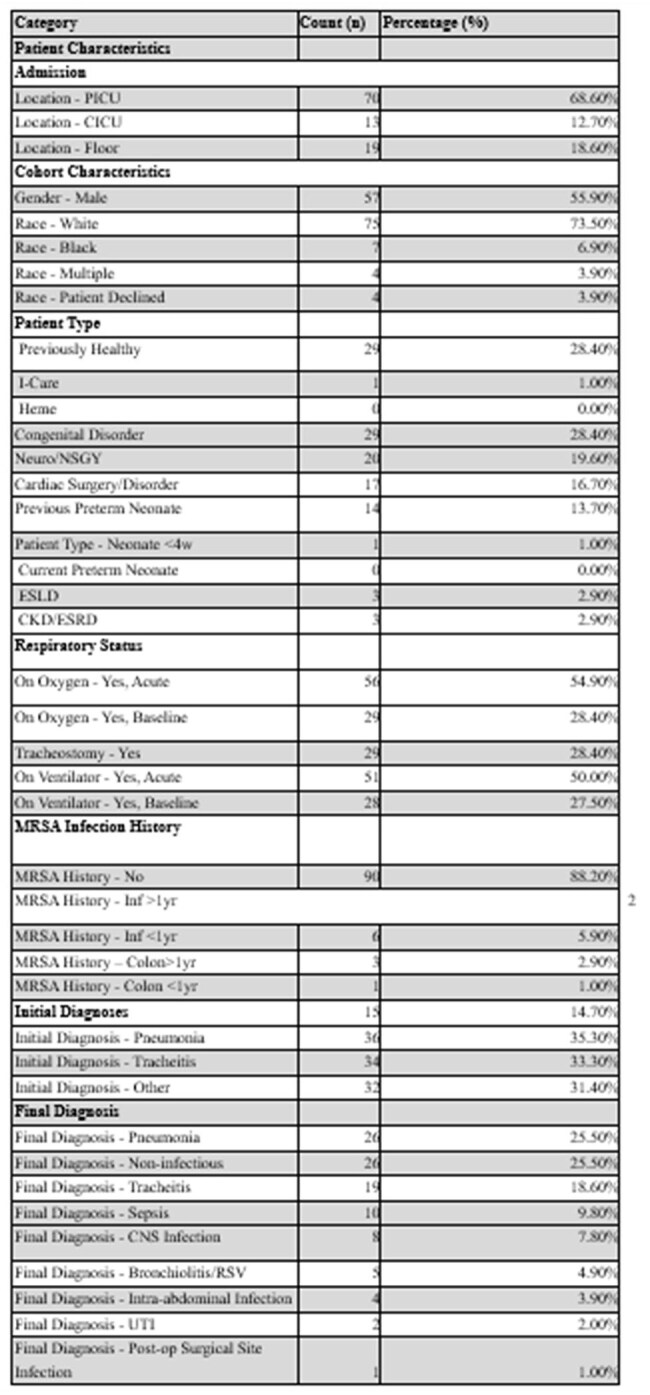
Patient Demographics and Clinical Characteristics

**Table 2.**
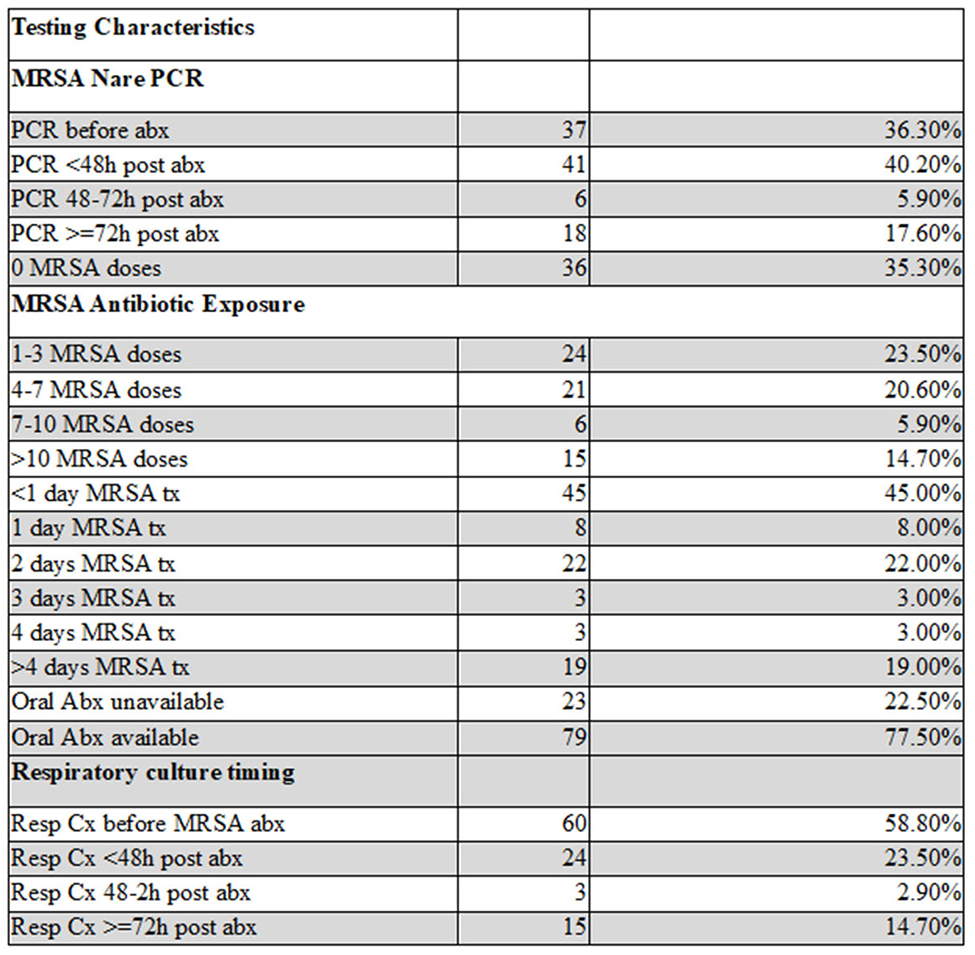
Diagnostic Performance of MRSA Nares PCR Testing

**Methods:**

We conducted a prospective, single-center study of hospitalized children (< 18 years) undergoing evaluation for LRTIs. MRSA nares PCR results were compared to lower respiratory tract cultures (LRTC), including tracheal, endotracheal, sputum, or BAL specimens. Immunocompromised or neutropenic patients were excluded. Informed consent was obtained. We calculated sensitivity, specificity, positive predictive value (PPV), and NPV of MRSA nares PCR in predicting MRSA from LRTC.

**Results:**

Among 102 patients (mean age 7.0 years; 55.9% male), 10 (9.8%) had positive MRSA nares PCR and 4 (3.9%) had culture-confirmed MRSA. Sensitivity and NPV were 100%, with no false negatives. Specificity was 93.9%, and PPV was 40%. Among 15 patients who received >72 hours of MRSA therapy before PCR testing, one had a positive PCR but all cultures were negative. Most patients (83.3%) required oxygen support. Mechanical ventilation was required in 50% for acute illness, and 27.5% were ventilated at baseline. Final diagnoses included pneumonia (25.5%), non-infectious conditions (25.5%), and tracheitis (18.6%). MRSA PCR was collected before antibiotics in 36.3% and ≥72 hours after antibiotics in 17.6%.

**Conclusion:**

MRSA nares PCR is a reliable rule-out test in pediatric LRTIs. Its high NPV supports early de-escalation of anti-MRSA therapy, including in critically ill patients. It offers value in antimicrobial optimization, particularly when cultures are delayed or unobtainable.

**Disclosures:**

Michael D. Green, MD, MPH, ADMA: Advisor/Consultant|Bristol Myers Squibb: Advisor/Consultant|ITB-MED: Advisor/Consultant Evan E. Facer, DO, AbbVie, Inc.: Grant/Research Support

